# Transcriptomic Signature of Spatial Navigation in Brains of Desert Ants

**DOI:** 10.1002/ece3.70365

**Published:** 2024-10-03

**Authors:** Luisa Maria Jaimes‐Nino, Adi Bar, Aziz Subach, Marah Stoldt, Romain Libbrecht, Inon Scharf, Susanne Foitzik

**Affiliations:** ^1^ Institute of Organismic and Molecular Evolution Johannes Gutenberg University Mainz Mainz Germany; ^2^ School of Zoology, George S Wise Faculty of Life Sciences Tel Aviv University Tel Aviv Israel; ^3^ Insect Biology Research Institute, UMR7261, CNRS University of Tours Tours France

**Keywords:** gene expression, learning, maze, path integration, step integration

## Abstract

Navigation is crucial for central‐place foragers to locate food and return to the nest. *Cataglyphis* ants are renowned for their advanced navigation abilities, relying on landmark cues and path integration. This study aims to uncover the transcriptomic basis of exceptional spatial learning in the central nervous system of *Cataglyphis niger*. Ants navigated a maze with a food reward, and we examined expression changes linked to correct decisions in subsequent runs. Correct decisions correlated with expression changes in the optic lobes, but not the central brain, showing a downregulation of genes associated with sucrose response and *Creb3l1*. The latter gene is homologous to *Drosophila* crebA, which is essential for long‐term memory formation. To understand how ants use distance information during path integration, we analyzed expression shifts associated with the last distance traveled. We uncovered a transcriptomic footprint in the central brain, but not in the optic lobes, with genes enriched for energy consumption and neurological functions, including neuronal projection development, synaptic target inhibition, and recognition processes. This suggests that transcriptional activity in the central brain is necessary for estimating distance traveled, which is crucial for path integration. Our study supports the distinct roles of different brain parts for navigation in *Cataglyphis* ants.

## Graphical abstract

Navigation mechanisms include path integration and landmark‐based navigation, and we show the first evidence for molecular pathways required for navigation in desert ants. Transcriptional activity in the central brain but not in the optic lobes might be necessary for the estimation of distance traveled, which is crucial for path integration. Correlation in gene expression and correctness in the optic lobes may indicate the importance of visual processing in route recognition.

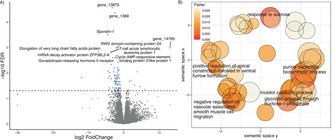

## 1 Introduction

Navigation is a fundamental ability of all central‐place foragers for finding food and homing, but also for retracing previously conducted outward journeys (Collet et al. 2020; Narendra 2021; Padget et al. 2017). The costs of foraging can be high, not only including time and energy spent but also the risk of predation, desiccation, or getting lost. Animals, from humans to insects, evolved sophisticated navigation skills to orientate themselves in space and also to direct their movement toward specific goals (Bingman and Cheng 2005; Collett and Collett 2000; Freas and Cheng 2022; Houston 2011; Muheim, Sjöberg, and Pinzon‐Rodriguez 2016).

One mechanism for navigation, called path integration, involves the neurological processing of sensory information to determine the direction and distance. Insects estimate the navigated distance by using the rate of information flow across the retina, known as optic flow (Mauss and Borst 2020; Pfeffer and Wittlinger 2016; Portelli et al. 2011) and/or a step integrator (Wittlinger, Wehner, and Wolf 2007, 2006), which can be particularly important in walking insects. Specifically, ants determine the direction of movement based on self‐motion information from their body senses, using cues such as the Earth’s magnetic field (Fleischmann et al. 2018; Grob et al. 2024), the polarization of the light, and the spectral pattern of the sky (Freas et al. 2024; Grob et al. 2021; Müller and Wehner 2007; Wehner and Müller 2006). Furthermore, they can rely on landmark navigation (e.g., visual, olfactory, magnetic, among other sensory cues) by aligning memories acquired on navigation journeys to the navigator's current view (Collett, Chittka, and Collett 2013; Freas and Spetch 2023; Zeil 2023). Since navigation mechanisms can be imprecise or prone to error (Merkle, Knaden, and Wehner 2006), ants perform systematic searches near their target location that can vary in structure and speed (Schultheiss and Cheng 2011; Wehner and Srinivasan 1981), such as changes in lateral oscillations or loops with increasing size and specific velocity (Clement, Schwarz, and Wystrach 2023; Schultheiss, Cheng, and Reynolds 2015).

In ants, the calibration of the sky compass and magneto‐sensory pathways, required for path integration, are processed in the central complex of the brain (Grob et al. 2024; Habenstein et al. 2020). Full memories from path integration may last only 24 h and decline thereafter (Cheng, Narendra, and Wehner 2006; Collett and Collett 2009). In contrast, recollections of visual landmarks (or other cues) may last for the entire lifetime of ant foragers (Narendra et al. 2007; Ziegler and Wehner 1997). The mushroom bodies in the insect brain store landmark information (Buehlmann et al. 2020; Heisenberg 1998; Hourcade et al. 2010; Mizunami, Weibrecht, and Strausfeld 1998; Webb and Wystrach 2016), but also serve as integration centers of sensory information (Kirkhart and Scott 2015; Strausfeld et al. 1998). The stored information can be used for orientation by alignment (Philippides et al. 2011; Stürzl and Zeil 2007), comparing the current sensory input with sensory information in the memory to produce a difference signal and generate a desired heading vector (Narendra, Gourmaud, and Zeil 2013; Wystrach, Beugnon, and Cheng 2012).

One of the best‐studied models for insect navigation is the desert ants of the genus *Cataglyphis*. These ants live in habitats that can reach extremely high temperatures and scavenge during the day when the risk of death from desiccation is considerable (Aron and Wehner 2021) and have developed sophisticated navigation skills. *Cataglyphis* workers forage solitarily over long distances (up to 100 m) and do not rely on pheromone‐based mass recruitment (Lenoir et al. 2009). They can navigate effectively through a maze under laboratory conditions using visual and olfactory cues (Gilad et al. 2023; Schatz et al. 1999; Chameron et al. 1998). Maze solving in *Cataglyphis niger* has been tested from a colony perspective. Three runs are sufficient for workers to improve their navigation through a binary‐tree maze by reaching the reward more quickly (i.e., a shorter discovery time, Bega et al. 2020; Saar et al. 2017) and with more correct navigational decisions (Bega et al. 2020). If experienced workers are removed, there is no improvement in discovery time or correct turns compared to the first run (Bega et al. 2020). This indicates that the optimizations in reaching the reward are due to experienced workers remembering the maze and navigating more efficiently in subsequent runs. Two weeks after the last training session in the maze, foragers take the same amount of time to reach the reward as naive ants, suggesting a loss of route memory (Saar et al. 2017).

The mechanism for distance estimation likely differs between flying and walking insects. The latter relies less on optic flow (Ronacher et al. 2000), and this mechanism seems to operate independently of the step integrator (Pfeffer and Wittlinger 2016; Ronacher et al. 2000; Ronacher and Wehner 1995). When *Cataglyphis fortis* ants walk on uneven terrain, the ants do not estimate the distance traveled but the horizontal displacement (or ground distance) (Wohlgemuth, Ronacher, and Wehner 2001). Additionally, when the leg length of these ants is manipulated, they miscalculate their traveled distance (Wittlinger, Wehner, and Wolf 2006, 2007), indicating that distance estimation in these desert ants does not rely on energy expenditure but rather on the integration of sensory cues. Proprioceptors in the body joints may be involved in step integration, but the details and molecular footprint of this process in the nervous system are still unknown. While the neurological and physiological foundations of optic flow have been studied, the mechanism of step integration and its molecular basis remain poorly understood.

Here, we aim to understand the transcriptional basis of spatial learning in the brains of *Cataglyphis niger* workers from an individual worker‐level perspective. To achieve this, we allowed workers to navigate through a maze to discover a food reward and tracked their navigation. In subsequent runs, the ants could use the learned spatial information to navigate toward the location where the reward was previously encountered. First, we analyzed the behavior of workers to identify those that exhibited a higher proportion of correct turns in later runs compared to those that did not improve their route. We expected these behavioral differences, which may indicate varying learning performances, to correlate with transcriptional activity in the brain. Correct turns are defined as those that bring the forager closer to the reward. As proxies for spatial memory of the maze, we considered the proportion of correct turns and the rate of navigation improvement, with the latter defined as the difference in the proportion of correct turns between runs. We expected gene expression in the central brain, including the central complex and mushroom bodies—areas that contain half of all brain neurons and exhibit structural neuronal plasticity (Grob et al. 2024), to be linked to of sensory information storage and processing.

We analyzed gene activity in the optic lobes separately, as this may be required for image matching when returning to the reward location. The retinal image of the current view should match the image previously stored in the mushroom bodies. Visual interneurons in the optic lobe, particularly in the lobula and lobula plate, are essential for visual matched filtering (Hausen 1982; Nordström and O'Carroll 2009; Schwind 1978), a process limiting the information that is transmitted and processed by the brain (Warrant 2016; Wehner 1987). Gene pathways whose expression is related to correct navigation through the maze are predicted to include those associated with memory formation and retention. In flies, this includes the cyclic AMP second messenger pathway in the mushroom bodies (Blum et al. 2009), the cAMP response element binding (CREB; Lin et al. 2021) and the S6 kinase II (S6KII) signaling pathway required for spatial orientation memory (Neuser et al. 2008). In both flies and honeybees, the dopaminergic pathway is involved in the motivational system of learning (Mancini et al. 2018; Waddell 2010), but its role in ants is as yet unclear.

The step integrator or *ant odometer* allows ant workers to predict approximately when they should have arrived near the nest by storing information about the distance already traveled (Wittlinger, Wehner, and Wolf 2006). To explore the potential transcriptional association behind the step integration process, we also analyzed whether gene expression in the brain is related to the last distance traveled in the maze. We expect that ants traveling longer distances would exhibit higher metabolic activity in the brain; however, other neurological mechanisms, independent of energy expenditure, may also be required for the step integration process. In honeybees, distance estimation correlates with the expression of taxon‐specific apidaecin genes, a box A‐binding factor, a transcriptional regulatory factor, and a chymotrypsin inhibitor in the mushroom bodies (Manfredini et al. 2023). However, a different mechanism may be expected in ants, as they do not primarily rely on optic flow for navigation.

## 2 Materials and Methods

### 2.1 Ant Collection and Colony Maintenance

**FIGURE 1:**
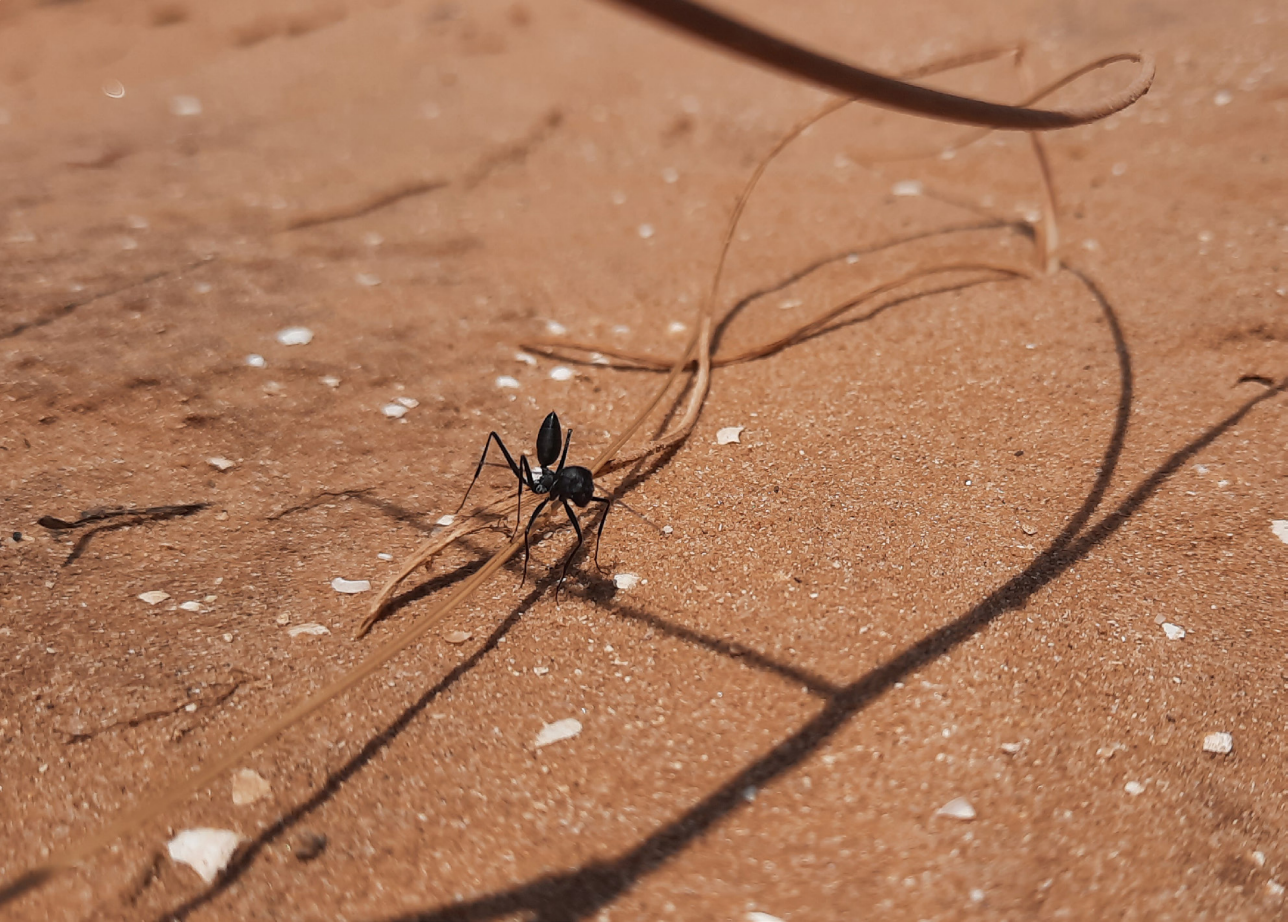
Worker of *Cataglyphis niger* in the Tel Baruch sand dunes near Tel Aviv. Credit to Adi Bar.

Eight *Cataglyphis niger* colonies (~100 workers each) were collected from the Tel Baruch sand dunes (32.1283 N, 34.7867 E) in January–September 2021 (Figure 1), transferred to the laboratory, and kept at ~25°C, 12:12 L:D in Plexiglas cages (50 × 20 × 5 cm). Around 2 weeks after collection, 50 worker ants of *C. niger* (no brood and no queen) per colony were separated and placed in an acclimation box. *C. niger* workers do not reproduce when reared in isolation (Aron, Mardulyn, and Leniaud 2016). The workers were randomly selected from diverse sizes and marked by gluing a paper sheet with a number (5 colors, numbered 0–9) to the thorax. Before the experiment started, the ants were starved for 10 days to increase foraging activity.

### 2.2 Spatial Exploration of the Maze

The maze presents a sequence of binary choices and repeating subunits with the same possible moves in each subunit, but only one route leading to the reward. The maze used is similar to those previously described (Bega et al. 2020; Saar et al. 2017, Figure 2A), but with the difference that the reward was placed in the inner chambers, requiring the ants to turn either left–right–left or right–left–right. Outer chambers were avoided to prevent the ants from “following walls,” which could make it easier for them to reach the outer chambers (e.g., right–right–right or left–left–left, Grüter et al. 2015). The acclimation box is set in front of the maze, and the cotton wool sealing the entry is removed. At the other side of the maze, an Eppendorf lid was placed with 0.5 g honey as a reward. The workers were allowed to search the maze for 50 or 30 min after the first worker reached the reward (whatever happens first). Subsequently, all workers that did not return to the acclimatation box by themselves were returned manually, and two more runs were performed after a 30‐min interval. The transcriptional response of immediate early genes is around 20 min, and genes related to learning can take a few hours to produce transcripts (Bahrami and Drabløs 2016; O'Brien and Lis 1993; Walton et al. 1999). We recorded the identity of all workers that arrived at the reward, the time they entered the maze for the first time, and their time of arrival. Additionally, we video‐recorded the whole run for in‐depth analyses of the navigation behavior. After the third run, all workers were shock‐frozen in liquid nitrogen and stored at −80°C.

**FIGURE 2:**
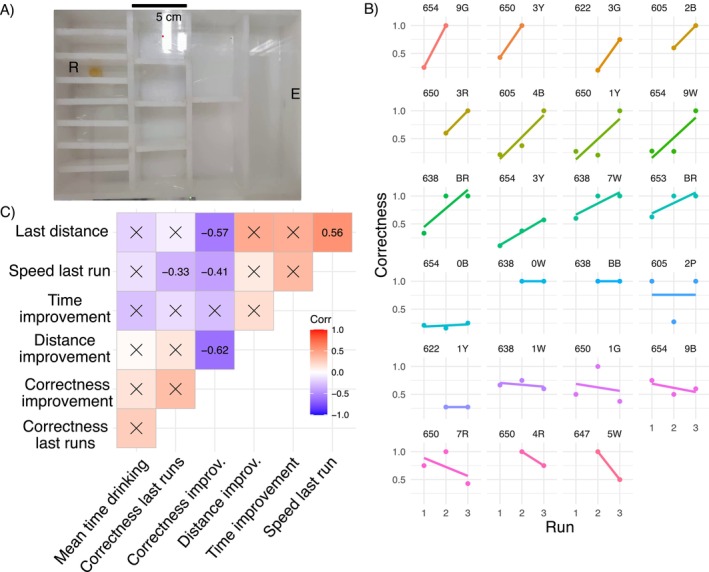
Maze and ant behavior. (A) Maze showing the entrance to the nest (E) from the acclimatization chamber and the chamber with reward (R). (B) The proportion of correct turns (correctness) to reach the reward (*n* = 23 individuals). (C) Correlation matrix of behavioral variables: Last distance and speed last run are the distance and average speed of an ants' last run; time improvement is the time difference reaching the reward between the last and the first run, proportional to the total time in the runs; distance improvement (*L*_dist_) and correctness improvement (*L*_prop_) refer to the change in the proportion of distance and correct turns over time, respectively; correctness last runs is the mean proportion of correct turns made in the second and third runs; mean time drinking is the average drinking time per run. Only statistically significant (*p* < 0.05) Pearson's correlations are shown.

### 2.3 Analysis of Navigation Behavior

In total, 51 workers reached the reward in at least two of the three runs. From this, a subset of 24 individuals was selected for further analysis and sequencing, based on highest variability across all workers in the proportion of time needed to discover the reward in the last run, compared to the first run (time improvement). The open software AnimalTA (v2.3.4, Chiara and Kim 2023) was used to manually track the route (1.88 fps) of the workers selected for sequencing through the maze. Their individual path was smoothed using the Savitzky–Golay filter in the program. For each worker and run, we noted the following behavioral variables (Tables S1 and S2): the distance and average speed to reach the chamber with the honey the first time in each run, the distance, and average speed in the complete run. In addition, the total time the honey was ingested and the time between the last food intake and sacrifice were recorded. Food intake was defined as the interaction of the mouthparts with the honey for more than three consecutive seconds, as a time below this threshold could be considered as tasting (antennation of the honey) but not necessarily feeding on the honey. For the following analysis, only individuals that fed on the reward were considered (*n* = 23) reducing the total colonies tested from eight to seven.

In terms of navigation in the maze, we tracked the number of wrong and correct turns made by each worker per run. Wrong or correct turns are defined here as forward movements (leftward through a door in Figure 2A) in the wrong direction to the reward's chamber or forward movements in the correct direction. The proportion of correct turns per run was calculated per individual until reaching the reward chamber and during the whole run (Corr_prop and Corr_compl, respectively, in Table S2). The latter includes information on the proportion of correct turns of unsuccessful runs (including decisions made between successful runs), because the ants gain experience by exploring the maze after finding the honey chamber in unsuccessful runs. As both variables were highly correlated (Pearson's correlation = 0.71, df = 21, *τ* = 4.61, *p* < 0.001), we kept the former as a proxy of efficiency when navigating the maze. Given that differences in time do not capture the nuances of the behavior (ants could stand still in the maze and groom themselves or enter back to the nest before reaching the reward), we decided to focus our analysis on the correctness of the turns as a proxy for learning the route to the reward, as it has commonly used (Grüter et al. 2015; Pasquier and Grüter 2016), rather than the time improvement.

We deduce a rate in navigation improvement (*L*_prop_) as the change in information with successive runs in the same maze as the difference in proportion of correct movements between runs: $L_{\text{last}-\text{first}}=\Delta \left(\frac{C}{C+W}\right)$ similar to previous calculations in a study using the same species (Bega et al. 2020). The first time a naive ant enters the maze, she will explore it without any prior knowledge of a reward, without inclination to reach any specific chamber, and is here defined as the training run. In contrast, the following times it enters the maze, we assume that the ant will be actively searching for the honey chamber, leading to an increase in the *L* rate, if the ant remembers the path or uses other cues to orient itself. We define the last run for each individual as the final time they reached the reward, which could be during the second or third test run, depending on the individual. The first run is defined as the first time the ant enters the maze and reaches the reward, which could be on the first or second run, depending on the individual. The rate of improvement in navigation is calculated between the first and last runs, based on the movements made from the start of the run until the honey chamber is reached. Similarly, a distance improvement (*L*_dist_) is calculated for the traveled distance in the last and first run.

### 2.4 Statistical Analysis of the Navigation Behavior

To describe the navigation process and select the variables of interest for expression analysis, we tested for possible correlations between the measured behavioral variables described above, using the function cor (Pearson correlation, R stats v.4.3.1), and cor_pmat from the ggcorrplot package (v. 0.1.4.1). To test if ants that improve in the proportion of correct turns across runs and move at a higher speed, we used a linear model (glmmTMB, v. 1.1.8). We expected that ants navigating a third time might show greater improvement compared to those navigating only twice, so we aimed to control for it by including the number of times the ant ran through the maze (either twice or three times) as a random factor. While it is clear that *Cataglyphis* ants do not use pheromones to recruit their nestmates to food sources, it is unknown whether they can perceive the footprints of their nestmates who have previously traveled through the maze, as has been demonstrated in *Lasius niger* (Wüst and Menzel 2017). To rule out the possibility that this influences the correct turns to reach the reward chamber, we tested whether the number of ants that reached the reward before them is linked to their navigation accuracy. Therefore, we tested the effect of the number of ants visiting the reward chamber before our tracked worker reached it on the proportion of correct turns of the tracked worker. Moreover, to rule out that the encounters with other ants affected the targets' correctness, we estimated the proportion of ants in a 2 cm radius of the tracked worker at a 10‐frame rate until reaching the reward and correlated it to the targets' correctness. The model's diagnostics were checked using DHARMa (0.4.6).

### 2.5 RNA Extraction, Sequencing, and Preprocessing of RNA‐Seq Data

The central brain, tissue including the antennal lobes and the mushroom bodies, and the two optic lobes tissue from the 23 individuals were dissected. Each of the two tissues was placed separately into 1.5 mL tubes containing 50 μL of Trizol and stored at −80°C. Total RNA was extracted after manual tissue homogenization with plastic pistils and using the Direct‐zol RNA miniprep Zymo following standard instructions. We obtained on average a total yield of 87 ng of mRNA from samples of the central brain and 59 ng from the optic lobes. Afterward, samples were sent to Novogene (Cambridge, UK) for RNA quality control, library construction, and sequencing at 150 bp paired‐end reads on an Illumina NovaSeq6000. After quality control, we sequenced 18 samples for the central brain and 15 for the optic lobes that resulted in an average of 30 Mio. reads and 25 Mio. reads, respectively (Table S3). Reads were adapter‐ and quality‐trimmed using TrimGalore (v. 0.6.7) and ran on the Galaxy platform usegalaxy.org (The Galaxy Community et al. 2022). Read quality was assessed using FastQC (v. 0.12.1) and summarized using MultiQC (v. 1.11.). Trimmed reads were mapped against the reference genome of *C. niger* (v. 3.1) (Cohen, Inbar, and Privman 2023) using STAR (genome‐mode, version 2.7.10b) on Galaxy using default parameters and specifying ‐‐quantMode GeneCounts, for later use in DESEq2 analysis. Back mapping of the samples was an average of 87.15% (average uniquely mapped transcripts, central brain: 84.80%, optic lobes: 89.94%, Table S3). We mapped the counts to 14,889 genes and filtered out genes with counts below 10 reads in at least six samples. Therefore, we kept 9583 genes expressed in the central brain 9622 genes in the optic lobes for further DESEq2 analysis (Tables S4 and S5).

### 2.6 Gene Expression Analysis

To model gene expression on brain transcriptional activity, we selected variables that could capture information on the navigation, such as the proportion of correct turns (measured as the average of proportion of correct turns in the test runs), and the correctness improvement, indicating changes in efficiency of the navigation after subsequent runs. We expected transcriptional activity in the brain to correlate to behaviors exhibited in the last performed run; therefore, we modeled the distance traveled in the last run before sampling. To avoid the results being heavily influenced by a few samples, we filtered potential outlier genes based on the maximum Cook's distance per gene and sample, using a Cook's cutoff of 6.55 for the central brain and 6.70 for the optic lobes samples (calculated by default following DESeq2 v. 1.40.2, Love, Huber, and Anders 2014). Therefore, 1051 genes expressed in the central brain and 882 in the optic lobes were filtered out and we compared full models that included either the last distance traveled, the correctness in the last runs and the correctness improvement as explanatory variables to reduced models without them using a likelihood ratio test (LRT) and FDR‐corrected *p*‐value < 0.05 as a significance threshold. A principal component analysis was performed with the samples using plotPCA. The functional annotations were obtained for *C. niger* (v. 3.1) (Cohen, Inbar, and Privman 2023) to perform Gene ontology (GO) enrichment analysis (topGO v. 2.52.0, Alexa and Rahnenfuhrer 2010), from the differentially expressed genes using weight01 algorithm and calculating the Fisher test. For visualization purposes, we summarize and remove redundant GO terms using Revigo (v.1.8.1, Supek et al. 2011), to report GO terms of the central brain expression data. Revigo finds a representative subset of the terms using a clustering algorithm that relies on semantic similarities.

## 3 Results

### 3.1 Navigation Behavior

Based on the time improvement in reaching the food reward between the first and the last run (divided by the total run time), we selected 23 ant workers from seven colonies for further analysis, representing the highest variability within the workers (exhibiting improvement, no change, or worsening in time). For these workers, we recorded the proportion of correct turns on the way to the reward chamber in their first, second, and third runs, if available (Figure 2B). Notice that ants can make more than three correct turns to reach the reward if the ant backtracks but continues to move forward in the correct direction. The time improvement did not correlate significantly to the correctness improvement (${\mathrm{cor}}_{\text{Time improvement}-L_{\text{prop}}}$ = −0.28, *p* = 0.10, Figure 2C), nor to the distance improvement (${\mathrm{cor}}_{\text{Time improvement}-L_{\text{dist}}}$ = −0.28, *p* = 0.10, Figure 2C); therefore, we focus on the correctness and distance improvement further on.

A significant increase in correct turns by 20% in the subsequent runs showed an improvement in navigation in the maze (glmmTMB, *Z*‐value = 3.15, *p* = 0.001). Workers that exhibited an increase in correct turns (positive values of *L*_prop_) over time also walked over shorter distances to reach the reward in subsequent runs (negative values of *L*_dist_, ${\mathrm{cor}}_{L_{\text{prop}}-L_{\text{dist}}}$ = −0.62, *p* < 0.014, Figure 2C). Thus, workers that made more correct navigational decisions took shorter routes. Yet, workers who made more correct turns navigated the maze slower in the final run (${\mathrm{cor}}_{\text{Correctness last runs}-\text{Speed last}\;\mathrm{run}}$ = −0.33, *p* = 0.02, Figure 2C). Ants that improved in their navigation moved shorter total distances in the last run (${\mathrm{cor}}_{L_{\text{prop}}-\text{Last distance}}$ = −0.57, *p* = 0.003, Figure 2C), but did so at lower speed (${\mathrm{cor}}_{L_{\text{prop}}-\text{Speed last}\;\mathrm{run}}$ = −0.41, *p* = 0.043, Figure 2C). Conversely, the longer the distance traveled by workers in the last run, the higher their speed (${\mathrm{cor}}_{\text{Last distance}-\text{Speed last}\;\mathrm{run}}$ = 0.56, *p* = 0.022, Figure 2C). Finally, we did not find a correlation between the average time drinking the reward and the proportion of correct turns in the test runs (${\mathrm{cor}}_{\text{Correctness last runs}-\text{Mean time drinking}}$ = 0.26, *p* = 0.29, Figure 2C), nor to the improvement (${\mathrm{cor}}_{\text{Correctness last runs}-L_{\text{prop}}}$ = 0.15, *p* = 0.34, Figure 2C).

**FIGURE A1:**
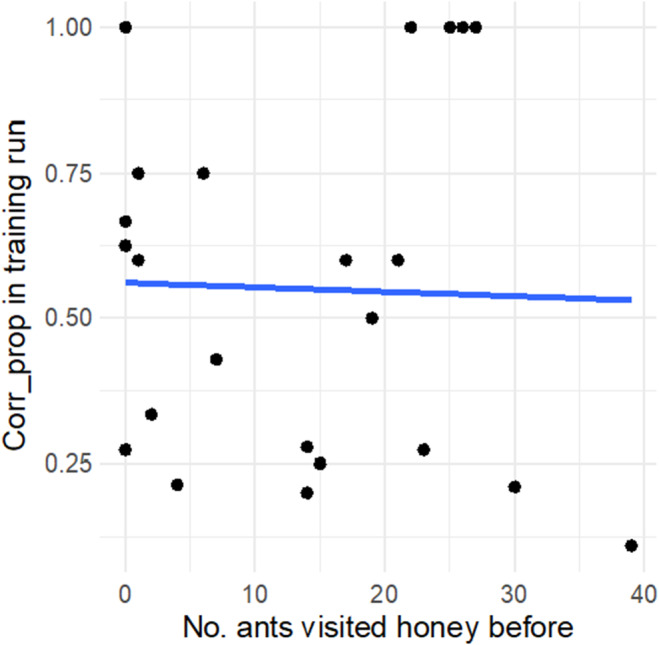
Relation between the number of ants that entered the reward chamber before the tracked individual in their first training run (*n* = 23) and correctness. The correctness is measured as the proportion of correct turns until the reward chamber is reached.

Interestingly, of the 23 tracked workers, seven reached the reward chamber first compared to the other seven chambers, which is more than chance would suggest (*X* ^*2*^ = 6.76, *p* < 0.01). We found weak evidence for a link, between the correctness in the first run and the correctness in subsequent runs (cor_Correctness training vs. Correctness last runs_ = 0.37, *p* = 0.08). Thus, naïve workers that reached the reward chamber with a high accuracy during the first run tended to do so also in subsequent runs. We wanted to rule out that workers were following CHC footprints of nestmates when first exploring the maze, as shown for other insects (Rottler, Schulz, and Ayasse 2013; Saleh et al. 2007; Wüst and Menzel 2017). We analyzed whether the number of workers that visited the reward chamber, before the tracked worker reached it, influenced her correctness. We found no such effect of the number of ants previously entering the chamber on the first run correctness (*n* = 23, glmmTMB, *Z*‐value = −0.14, *p* = 0.89, Figure A1 in Appendix). We also investigated the possibility that naïve ants have been influenced by the decisions of other ants. We showed that the correctness of the first run did not depend on the proportion of encounters with other nestmates (*n* = 46, glmmTMB, *Z*‐value = 0.81, *p* = 0.42), nor an interaction between the encounters and the type of run (first or last run × Encounters, glmmTMB, *Z*‐value = −0.217, *p* = 0.83).

### 3.2 Gene Expression in the Central Brain

**FIGURE 3:**
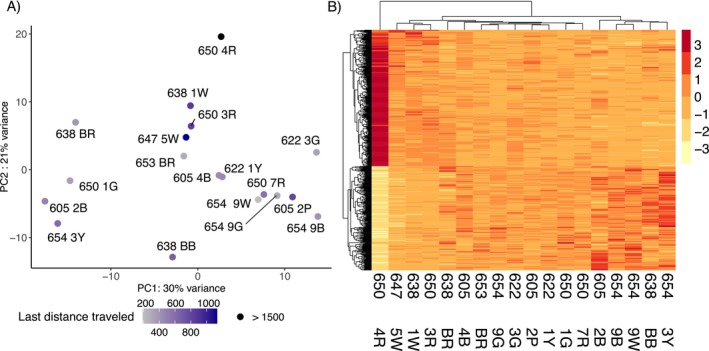
Central brain expression for the last distance traveled. (A) PCA of the variance transformed transcriptomic reads of central brain expression of 19 *Cataglyphis niger* workers. In total, 51% of the variance in expression profiles are represented by the first two axis of the PCA. The gray‐blue corresponds to the scaled and centered traveled distance (cm) in the maze in the last run before sacrifice. In black distances superior to 1500 cm are depicted. (B) Heat map of the normalized expression of DEGs in the central brain. *Z*‐scores are computed on a gene‐by‐gene basis by subtracting the mean and then dividing by the standard deviation after the clustering.

**FIGURE 4:**
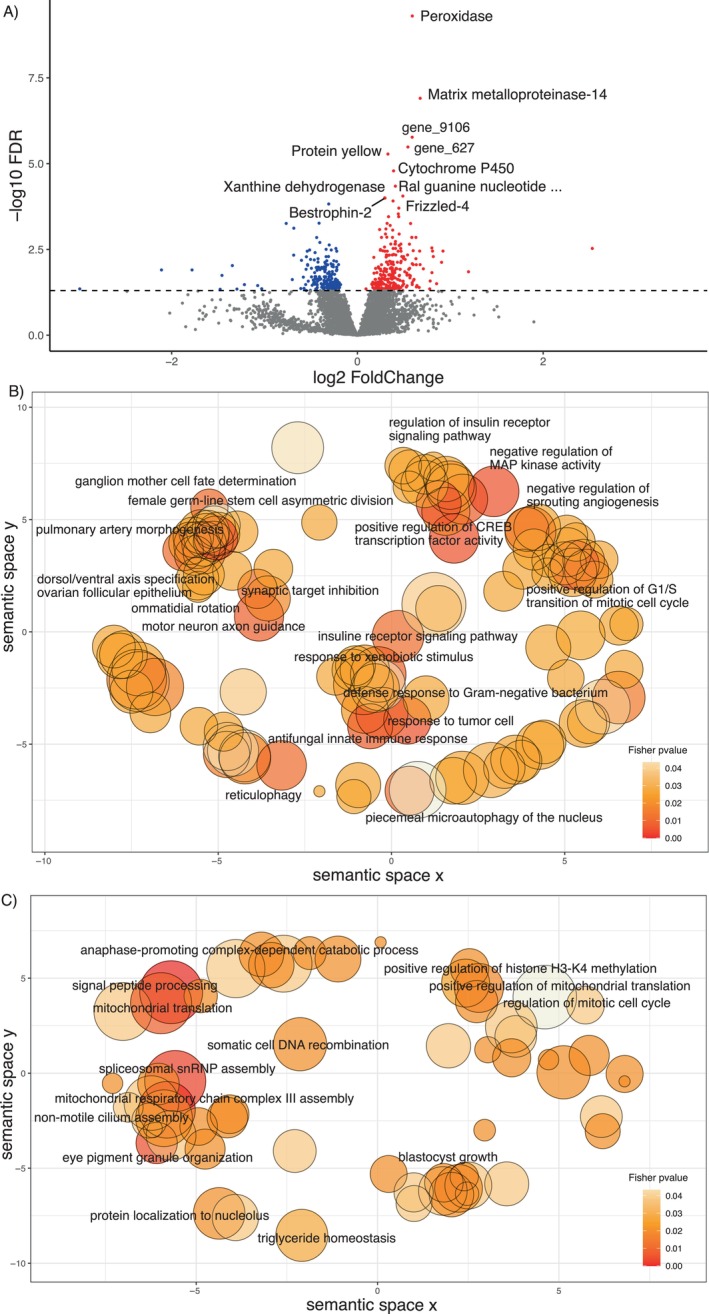
Gene expression in the central brain based on the distance traveled in the last run. (A) Log‐fold change in upregulated genes (red) and downregulated genes (blue) in ants walking larger distances. The 10 most significant genes are named. The horizontal dashed line represents the significance threshold of our differential expression analysis *p* < 0.05. (B) Representative subset of enriched GO terms in the central brain. Revigo visualization and clustering are based on the semantic similarities and hierarchical structure (parent–child terms) of GO terms. One single representative GO term enriched in workers that walked longer distances in the last run is shown for each cluster, with *p* < 0.05, and represented by more than one gene. The most statistically significant (and nonredundant) 19 upregulated GOterms and, (C) 14 downregulated DEGs GOterms. The bubble size is relative to the number of annotations for the GO Term ID in the underlying EBI GOA database.

The two principal components of the PCA of the central brain of 18 individuals (both with *λ* > 1) explained 51% of the total variation in gene expression (Figure 3A). No grouping pattern was noticeable based on colony identity (Figure 3B). No differentially expressed genes were found in response to the proportion of correct turns made during the test run(s) and correctness improvement in the central brain using DESeq2. However, we identified 478 DEGs, 271 up and 207 downregulated genes specifically (Figure 4A and Table S6), whose expression was better explained in a model with the distance moved in the maze during the last run. Among the 10 most significant DEGs, whose expression increased with increasing walking distance, we found genes involved in oxidative processes, such as cytochrome P450, peroxidase, and xanthine dehydrogenase. Other upregulated genes play a role in muscle activity, for example, matrix metalloproteinase‐14 and frizzled‐4, and the gene Bestrophin‐2, that encodes a calcium‐activated chloride channel (George et al. 2023). The GO term analysis showed enrichment in upregulated genes for 145 GO terms (74 represented by more than one annotated gene, Table S7 and Figure 4B). Central brain transcriptomes of ants moving over larger distances in the last run were enriched for processes related to energy intake and consumption, such as the “insulin receptor signaling pathway,” “cholesterol efflux,” “cholesterol homeostasis,” and “trehalose metabolism.” We also found enrichment for muscle activation processes such as “motor neuron axon guidance,” “response to muscle stretch,” “actin cytoskeleton reorganization,” “regulation of smooth muscle cell migration,” “regulation of actomyosin contractile ring contraction,” “muscle attachment,” and the “regulation of cardiac muscle contraction.”

Additionally, we uncovered pathways related to the neurological activity of the brain such as “neuron projection development” with 26 DEGs out of 461 annotated genes, for example, Serine/threonine‐protein kinase MARK1, Discoidin domain‐containing receptor A, Histone lysine acetyltransferase CREBBP, and Protein spaetzle encoding genes. The GO terms “synaptic target inhibition and recognition,” represented by the Extracellular sulfatase SULF‐1 homolog and Protein toll encoding genes, and “neurotransmitter receptor transport, postsynaptic endosome to lysosome” represented only by the gene AP‐3 complex subunit delta‐1 were enriched. Other enriched processes as the “ganglion mother cell fate determination,” “positive regulation of axon regeneration,” “axon ensheathment in central nervous system,” and “ommatidial rotation” are worth mentioning. Furthermore, we found enrichment in processes regulating the transcription such as “positive regulation of CREB transcription factor activity” represented by the Histone lysine acetyltransferase CREBBP and the CREB‐regulated transcription coactivator 3 encoding genes.

Downregulated genes in workers moving over larger distances included genes encoding the *Transcription factor IIIB 90 kDa subunit*, *Surfeit locus protein 1*, and the Post‐GPI attachment to proteins factor 3. GOterm of downregulated genes (99 GO terms, 35 represented by more than one gene) exhibit enrichment in “signal peptide processing,” “mitochondrial respiratory chain complex III assembly,” “triglyceride homeostasis,” “anaphase‐promoting complex‐dependent catabolic process,” and “eye pigment granule organization” processes, among others (Table S8 and Figure 4C).

### 3.3 Gene Expression in the Optic Lobes

**FIGURE 5:**
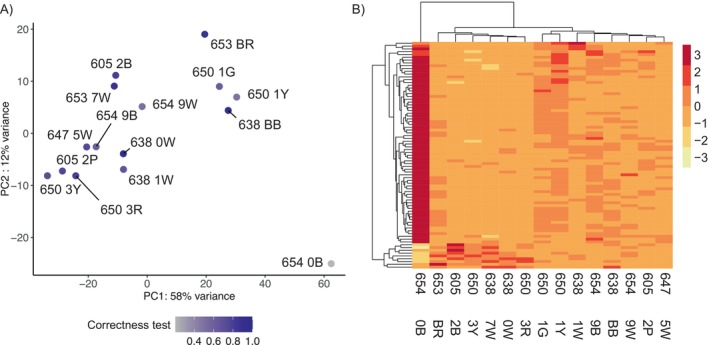
Optic lobe expression for the correctness test. (A) PCA of the variance transformed transcriptomic reads of the optic lobe's expression of 15 workers. (B) Heat map of the normalized expression of DEGs in the optic lobes. The gray‐blue corresponds to the average of the correct proportion of movements made in the maze in the test(s) run. *Z*‐scores are computed on a gene‐by‐gene basis by subtracting the mean and then dividing by the standard deviation after the clustering.

**FIGURE 6:**
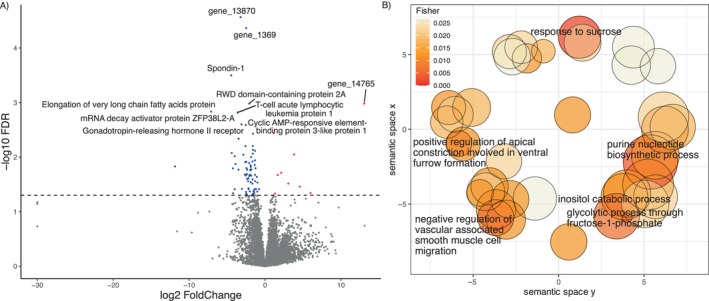
Optical lobe gene expression in *Cataglyphis niger* workers. (A) Log‐fold change in upregulated genes (red) and downregulated genes (blue) in ants exhibiting higher correctness in last runs. The 10 most significant genes are named. The horizontal dashed line represents the significance threshold of our differential expression analysis *p* < 0.05. (B) Revigo semantic clustering of the five most statistically significant (and nonredundant) GO terms with *p* < 0.05 for downregulated DEGs. The bubble size is relative to the number of annotations for the GO Term ID in the underlying EBI GOA database.

The first two axes of the PCA of the optic lobes of 15 individuals with *Λ* > 1 explained 73% of the total gene expression variation (Figure 5A). No grouping pattern was apparent based on colony identity (Figure 5B). We identified 81 DEGs (9 up and 72 downregulated genes, Table S9), whose expression was better explained in a model with the average proportion of correct turns made during the test(s) runs (Figure 6A). The upregulated genes include the Protein O‐mannosyl‐transferase TMTC2, Glutamate receptor ionotropic, Peroxynitrite isomerase THAP4, Ovarian‐specific serine/threonine‐protein kinase Lok, and the Carboxylesterase 5A. No functional annotation was found for four of the upregulated DEGs (gene_5625, gene_12173, gene_14297, and gene_14765). GO term analysis showed enrichment based only on a single annotated gene for the “response to glycoside,” “positive regulation of anoikis,” “cellular response to bisphenol A,” and “nitrate metabolic process” (Table S10).

Among the most significant downregulated genes, we found the RWD domain‐containing protein 2A, Gonadotropin‐releasing hormone II receptor, mRNA decay activator protein ZFP36L2‐A, Spondin‐1, Elongation of very long chain fatty acids protein 2, T‐cell acute lymphocytic leukemia protein 1, and the Cyclic AMP‐responsive element‐binding protein 3‐like protein 1 (Creb3l1). GO term enrichment for more than one annotated gene in downregulated DEGs occurred for the “response to sucrose,” “purine nucleotide biosynthetic process,” among other 41 enriched GOterms (Table S11 and Figure 6B). No differentially expressed genes were found in response to improvement and the distance walked in the last run in the optic lobe samples using DESeq2.

## 4 Discussion

Organisms that navigate can use a combination of path integration and landmark cues from their spatial memory to return to a previously known location. In our experiment, we allowed ant workers to discover a reward and to return to it in subsequent runs. Naïve ants located a honey reward in a bifurcation maze and with time improved navigation through this complex spatial set‐up. This improvement corresponded to an increase in the proportion of correct turns made and in a shorter path through the maze to reach the reward. Contrary to expectations, we found that the more the workers improved their route, the slower they walked through the maze. This could mean that the ants need to walk more slowly to find the right path, possibly to match their memory with spatial relationships or landmarks. We also found that the workers were unexpectedly likely to find the right chamber with the food reward at first try. However, based on our data, this was not due to following footprints or communication between ants, but possibly because the ants can follow the odor of the honey. Thus, our behavioral observations indicate that these desert ants may rely on a combination of olfactory cue tracking and spatial learning to improve their accuracy over time.

We detected for the first time in ants, an association between the gene expression in the brain and optic lobes and navigation behavior. We identified 478 DEGs in the transcriptomes of the brain whose expression correlated with the distance traveled during the last run. Some of these genes may be required for the step integration that takes place in the ants' mushroom bodies (Kirkhart and Scott 2015), which are part of the central brain analyzed here. While we did not find such a transcriptional footprint of walking distance in the optic lobes, we identified 81 DEGs associated to the correctness of turns in the last runs. These DEGs can provide information about the possible mechanisms that ants use for more efficient navigation, including image matching. We found no correlation between gene expression and correctness improvement. This could be due to the challenges of detecting patterns based on continuous responses, and/or a larger sample size might be required to observe such effects.

The genes that were overexpressed in the central brain of workers who walked longer distances were related to functions associated with energy metabolism, such as the insulin pathway, trehalose metabolism, and processes associated with muscle activity. These processes are expected to be related to locomotory activity. Among the neurological processes, we found enrichment of the motor neuron axon guidance, synaptic target, and neurotransmitter receptor transport. This is interesting as the physiological mechanisms of the step integrator are not well understood but might relate to proprioceptors in the muscles and joints of ants. Such processes can be expected to be required for step integration. Genes involved in immune defense mechanisms were also enriched in processes such as the antifungal innate immune response, response to xenobiotic stimulus, and defense response to Gram‐negative bacterium, among others. *Drosophila* flies require an antimicrobial peptide (diptericin) for the formation of long‐term memory associated with foraging behavior (Barajas‐Azpeleta et al. 2018). Furthermore, apidaecin seems to play an important role in distance perception and learning in the mushroom bodies of honeybees (Manfredini et al. 2023). It is known that persistent neurological activity is observed for path integration in flies, occurring in the ring attractor of the central brain, being a potential substrate for the retention of short‐term orientation memory when landmarks are temporally out of sight (Seelig and Jayaraman 2015). Taken together, these different lines of evidence suggest possible that immune peptides are required in the brain of insects for the process of distance assessment.

The downregulated processes are also of interest because the ants that took shorter routes in the last run showed the most improvements in their navigation. Triglyceride homeostasis and the processing of signaling peptides are particularly strongly represented among the downregulated genes. In mice, triglycerides have been shown to mediate cognitive impairment, likely through their ability to alter the release of feeding peptides, and that lowering triglycerides can reverse cognitive impairment and improve oxidative stress in the brain (Cansell and Luquet 2016; Farr et al. 2008). Additionally, overfed flies that exhibited hyperlipidemia downregulate many learning and memory‐regulating genes in the brain (Zhang et al. 2015). Thus, preserving the triglyceride homeostasis might be needed for learning and recalling previously visited routes.

In the optic lobes, we expected to find changes in gene expression that could be related to image matching of the learned route. It is important to consider that view‐based long‐term memory formation needs, depending on the desert ant species, more or less trips to occur. *Cataglyphis velox*, needs up to five experiences of the route to become established, with one experience being insufficient for long‐term memory formation (Freas and Spetch 2019). Here, ants are allowed to make two to three trips. While it is unknown if these are sufficient trials to fully establish long‐term visual memories, we did find a 20% general improvement in correct turns. We found a correlation between the gene expression and the proportion of correct turns in the last runs, for example, upregulation of the Glutamate receptor ionotropic and downregulation of the *Creb3l1*. The glutamate ionotropic (ion channel coupled) receptor contributes to neuronal communication and signal processing, which is finally needed for learning and memory formation in animals (Riedel, Platt, and Micheau 2003). Related to this, we encountered enrichment in the central brain for the positive regulation of CREB transcription factor activity of workers that walked larger distances. These workers also tended to be the less correct in the last run. The *Creb3l1* gene is involved in the dopaminergic synapse pathway in humans (Zhang et al. 2017) and in mammals it controls the motivation and reward, learning and memory (Sun et al. 2019). In *Drosophila*, the CREB3L1 homolog *crebA* (Kent and Agrawal 2020), and the paralog *crebB* genes encode CREB family proteins involved in long‐term memory formation (Lin et al. 2021). CREBA is overexpressed in the fly neurons up to a day after several training sessions of olfactory negative conditioning in a T‐maze, and its knockdown leads to impairment of long‐term memory formation. Contrary to our expectation, we found a downregulation of *Creb3l1* in ants that took the more correct route to the reward.

Moreover, we observed an upregulation of the response to glycoside, and downregulation of the response to sucrose, and the glycolytic process through fructose‐1‐phosphate on more correct ants. The fructose‐1‐phosphate inhibits the glycogenolysis (the breakdown of glycogen into glucose). In flies, glycolysis in the mushroom bodies is fundamental for the olfactory memory formation (Alberini et al. 2018; Wu et al. 2018), while in our results this process was found in the optic lobes. Given that ants that were correct in the last run also walked slower, the ants' production of glucose via glycogenolysis could be explained as the fuel needed to recall the path. In terms of honey consumption, we did not find a correlation of correctness with the time drinking honey. Nevertheless, the actual consumption of honey might vary in the individuals as they performed trophallaxis during the experiment.

Finally, our results support the hypothesis that ants are attracted to the smell of the honey. This indicates that there may be other processes involved (other than learning) that allow the ants to be effective at solving the maze, as illustrated by naive ants. Further experiments should test for this possibility and if needed use an inodorous reward. We found a general improvement in navigation that did correlate weakly with a higher correctness during the last runs. This could be due to workers that start with a high score, leaving little room for improvement. An inodorous reward will probably lead to more striking patterns. Lastly, ants that do not improve generally walked longer total distances in the last run, implying that after finding the reward they continue exploring the maze, compared to ants that improved. It is possible that those ants that exhibit a low spatial orientation continue searching for a reward afterwards.

## 5 Conclusion

In conclusion, our study sheds light on the transcriptomic associations underlying navigation in desert ants and reveals an interplay between the use of orientation cues and spatial learning. Naïve ants showed a remarkable ability to improve their navigation skills over time, as evidenced by greater correctness in maneuvering the maze and a shorter route to the reward. Surprisingly, this improvement in navigation was accompanied by a decrease in walking speed, suggesting a trade‐off between accuracy and speed in the ants' foraging behavior. Our investigation of gene expression patterns associated with distance traveled revealed that upregulated genes in the central brain are involved in energy metabolism and synaptic transmission, while downregulated genes point to the importance of triglyceride homeostasis and signal peptide processing, processes that could be involved in learning and memory consolidation.

In addition, the observed correlation between gene expression in the optic lobes and correctness in maze navigation may indicate the importance of visual processing in route recognition. However, the downregulation of genes associated with sucrose response and glycolytic processes in more correct ants raises questions about the metabolic requirements of navigation and the role of glucose metabolism in memory formation. Overall, our study provides the first evidence for molecular pathways required for navigation in desert ants and could lead to future research on the functioning of complex behaviors such as path integration and landmark‐based navigation. Further studies should focus on the transcriptional differences in more specific tissues (e.g., mushroom bodies and the central complex), given the current knowledge of specific processes occurring in different tissues.

## Author Contributions

**Luisa Maria Jaimes‐Nino:** data curation (equal), formal analysis (equal), validation (equal), visualization (equal), writing – original draft (equal), writing – review and editing (equal). **Adi Bar:** conceptualization (equal), data curation (equal), investigation (equal), methodology (equal), writing – review and editing (equal). **Aziz Subach:** conceptualization (equal), methodology (equal), project administration (equal), writing – review and editing (equal). **Marah Stoldt:** data curation (equal), formal analysis (equal), writing – review and editing (equal). **Romain Libbrecht:** conceptualization (equal), funding acquisition (equal), supervision (equal), writing – review and editing (equal). **Inon Scharf:** conceptualization (equal), funding acquisition (equal), investigation (equal), project administration (equal), supervision (equal), writing – review and editing (equal). **Susanne Foitzik:** conceptualization (equal), funding acquisition (equal), methodology (equal), project administration (equal), supervision (equal), writing – review and editing (equal).

## Ethics Statement

Animal care was in accordance with institutional guidelines. The experiments followed the rules of the animal protection law (Tierschutzgesetz), and official approvals were not necessary (no CITES species).

## Conflicts of Interest

The authors declare no conflicts of interest.

## Supporting information


Data S1.

Tables S1–S11.

## Data Availability

All other data are included in the manuscript and/or Tables S1–S11. The code is available in Data S1. Raw sequence reads are deposited in the SRA (BioProject PRJNA1104779 Desert ant navigation).
