# Supplementary material for: Transcriptomic Signature of Spatial Navigation in Brains of Desert Ants
Source: Ecol Evol. 2024 Oct 3;14(10):e70365. doi: 10.1002/ece3.70365 (PMC11449808; doi:10.1002/ece3.70365)
Supplement: Supplementary file 1 — Data S1. [file ECE3-14-e70365-s002.docx]

### Transcriptomic signature of the spatial navigation and step integration in desert ants

# **Authors:**Jaimes-Nino, Luisa Maria^1^; Bar, Adi^2^; Subach, Aziz^2^; Stoldt, Marah^1^; Libbrecht, Romain^3^; Scharf, Inon^2^; Foitzik, Susanne^1^

library(openxlsx)

library(ggcorrplot)

#### Correlations Fig.1B ####

indepVar <- read.xlsx("~/Data/Supl_tables.xlsx", sheet = 2)

indepVar$colony <- substr(indepVar$Ant_ID, 1,3)

rownames(indepVar) <- paste("CB_",indepVar$Ant_ID, sep="")

indepVar <- indepVar[-c(4),] # Remove ant 617_C_7Y that did not drink the food reward in any run.

cor.test(indepVar$Corr_compl, indepVar$Corr_prop_mean)

# Pearson's product-moment correlation

#

# data: samples_variables$Corr_compl and samples_variables$Corr_prop

# t = 4.6117, df = 21, p-value = 0.0001506

# alternative hypothesis: true correlation is not equal to 0

# 95 percent confidence interval:

# 0.4199275 0.8678065

# sample estimates:

# cor

# 0.7093455

sumvariables <- as.data.frame(cbind(Time_diff_prop = as.numeric(indepVar$first_vs_last_prop_v2), # Difference in time

Corrprop_test_mean = indepVar$Corrprop_test_mean,

Lprop= indepVar$Lprop,

# SpeedBefH_last = samples_variables$Speed_befh_last,

Aver_TimeDrinking = indepVar$TotalTimeDrinking,

Speed_LastRun = indepVar$Average_Speed_last,

Last_dist = indepVar$Traveled_Dist_last,

Ldist = indepVar$Ldist))

corr_matrix <- cor(sumvariables)

p.mat <- cor_pmat( corr_matrix)

ggcorrplot(corr_matrix, hc.order = TRUE, type = "upper", p.mat = p.mat, lab = T, sig.level = 0.049999)

cor.test(samples_variables$Corr_compl, samples_variables$Corr_prop)

#### First viisted chamber ####

# Probability of visiting the chamber of the reward first compared to any other chamber

chisq.test(c(7,16), p =c(1/8, 7/8))

first_chamber <- as.factor(c("h", "h", "1", "h",

"4", "5", "h", "4", "h",

"h", "2", "2", "1", "8",

"6", "1", "7", "7", "3",

"h", "1", "1", "1"))

first_chamber_ID <-c( "1", "2", "3", "4", "5" , "6", "7", "8", "h")

first_chamber_ants <-c( 6, 2 , 1, 2, 1 , 1, 2, 1, 7)

chamber_visited_first <- as.data.frame(cbind(first_chamber_ID, first_chamber_ants))

chamber_visited_first$first_chamber_ants <- as.numeric( chamber_visited_first$first_chamber_ants)

#### Central Brain Gene expression ####

library(openxlsx)

library(ggplot2)

library(dplyr)

library(patchwork)

library(ggcorrplot)

library("factoextra")

library(tidyverse)

library(DESeq2)

library(ggrepel)

centralbrain <- read.xlsx("~/Data/Supl_tables.xlsx", sheet = 4, header = T)

centralbrain_counts <- centralbrain[-c(1:2),] # Remove headers

colnames(centralbrain_counts) <- c("Gene_ID" , "CB_605_C_2B", "CB_605_C_2P", "CB_605_C_4B", "CB_617_C_7Y", "CB_622_C_1Y", "CB_622_C_3G", "CB_638_C_1W", "CB_638_C_BB", "CB_638_C_BR", "CB_647_C_5W", "CB_650_C_1G", "CB_650_C_3R", "CB_650_C_4R", "CB_650_C_7R", "CB_653_C_BR", "CB_654_C_3Y", "CB_654_C_9B", "CB_654_C_9G", "CB_654_C_9W")

rownames(centralbrain_counts) <- centralbrain_counts$Gene_ID

# Drop sample "CB_617_C_7Y", never drunk honey

centralbrain_counts <- centralbrain_counts[,-c(4)]

indepVar <- indepVar[, -c(32)] #remove the ant_id

colnames(indepVar) # "first_Corr_compl" is the first time the ant enters the maze

# "Corr_firstT" is the first time the ant is tested, second time it reaches the honey

# Select the data from the 18 samples sequenced for the central brain

indepVar_19 <- indepVar[rownames(indepVar) %in% colnames(centralbrain_counts),]

#Order the rows of the design data in the same order as the columns on the count data

colnames(centralbrain_counts)

rownames(indepVar_19)

# Center and scale variables

# Include the traveled distance from the last run

indepVar_19$sTraveled_Dist_last <- as.vector(scale(indepVar_19$Traveled_Dist_last, center = TRUE, scale = TRUE))

indepVar_19$Traveled_Dist_last1 <- "> 1500"

indepVar_19$Traveled_Dist_last1[indepVar_19$Traveled_Dist_last <= 1500] <- NA

dds_cenbrain <- DESeqDataSetFromMatrix(countData = centralbrain_counts, colData = indepVar_19 , design = ~ sTraveled_Dist_last )

keep <- rowSums(counts(dds_cenbrain) >= 10) >= 6

dds_cenbrain <- dds_cenbrain[keep,]

# Testing the last distance traveled in the central brain expression #

# A reduced model without the the last distance traveled

dds_cenbrain_ltr_Traveled_Dist_last <- DESeq(dds_cenbrain, test="LRT", reduced = ~ sTraveled_Dist_last )

LRT_results_Traveled_Dist_last <- results(dds_cenbrain_ltr_Traveled_Dist_last)

LRT_results_Traveled_Dist_last_sig <- subset(LRT_results_Traveled_Dist_last , padj < 0.05)

LRT_results_Traveled_Dist_last_sig # 634 only last distance term

# cooks filtering #

mcols(dds_cenbrain_ltr_Traveled_Dist_last)$maxCooks <- apply(assays(dds_cenbrain_ltr_Traveled_Dist_last)[["cooks"]], 1, max)

cooksCutoff <- qf(.99, 2, 18 - 2)

sum(mcols(dds_cenbrain_ltr_Traveled_Dist_last)$maxCooks > cooksCutoff, na.rm = T) #1051

LRT_results_Traveled_Dist_last$padj[mcols(dds_cenbrain_ltr_Traveled_Dist_last)$maxCooks > cooksCutoff] <- NA

LRT_results_last_dist_cooks_sig <- subset(LRT_results_Traveled_Dist_last , padj < 0.05)

LRT_results_last_dist_cooks_sig # 478 DEGs

summary(LRT_results_last_dist_cooks_sig)

#visualization

# DESeq2 creates a matrix when you use the counts() function

## First convert normalized_counts to a data frame and transfer the row names to a new column called "gene"

normalized_counts <- counts(dds_cenbrain_ltr_last_dist, normalized=T) %>%

data.frame() %>%

rownames_to_column(var="gene")

### Extract normalized expression for significant genes from the OE and control samples (2:4 and 7:9)

norm_OEsig <- normalized_counts %>%

dplyr::filter(gene %in% LRT_results_last_dist_cooks_sig$gene)

### Set a color palette

heat_colors <- RColorBrewer::brewer.pal(6, "YlOrRd")

### Run pheatmap using the metadata data frame for the annotation

pheatmap::pheatmap(norm_OEsig[2:19],

color = heat_colors,

cluster_rows = T,

show_rownames = F,

border_color = NA,

fontsize = 10,

scale = "row",

fontsize_row = 10,

height = 20)

LRT_results_last_dist_tb <- LRT_results_last_dist %>%

data.frame() %>%

rownames_to_column(var="gene") %>%

as_tibble()

LRT_results_last_dist_tb <- LRT_results_last_dist_tb %>%

dplyr::mutate(threshold_OE_pos = padj < 0.05

& log2FoldChange > 0)

LRT_results_last_dist_tb <- LRT_results_last_dist_tb %>%

dplyr::mutate(threshold_OE_neg = padj < 0.05

& log2FoldChange < 0)

# Now which is positive and which is negative

# LRT_results_last_dist_tb$threshold_OE <- cbind(LRT_results_last_dist_tb[,8:9], col = names(LRT_results_last_dist_tb[,8:9])[max.col(LRT_results_last_dist_tb[,8:9])])

LRT_results_last_dist_tb <- LRT_results_last_dist_tb %>%

mutate(color = case_when(threshold_OE_neg ~ "Negative",

threshold_OE_pos ~ "Positive"))

## Create an empty column to indicate which genes to label

LRT_results_last_dist_tb <- LRT_results_last_dist_tb %>% dplyr::mutate(genelabels = "")

## Sort by padj values

LRT_results_last_dist_tb <- LRT_results_last_dist_tb %>% dplyr::arrange(padj)

## Populate the genelabels column with contents of the gene symbols column for the first 10 rows, i.e. the top 10 most significantly expressed genes

LRT_results_last_dist_tb$genelabels[1:10] <- as.character(LRT_results_last_dist_tb $gene[1:10])

ggplot(LRT_results_last_dist_tb) +

geom_point(aes(x = log2FoldChange, y = -log10(padj), colour = color), size = 0.5) +

geom_text_repel(aes(label = genelabels, x = log2FoldChange, y = -log10(padj))) +

xlab("log2 FoldChange") +

ylab("-log10 FDR") +

geom_hline(yintercept =1.30103, linetype = "dashed")+

scale_color_manual(values = c("blue", "red", "grey")) +

theme_bw(legend.position = "none",

plot.title = element_text(size = rel(1.5), hjust = 0.5),

axis.title = element_text(size = rel(1.25)))

# PCA plot

# For plotting

dds_vst_cenbrain <- varianceStabilizingTransformation(dds_cenbrain)

expression_vst_cenbrain <- assay(dds_vst_cenbrain)

pca_transf <- FactoMineR::PCA(centralbrain_counts)

summary(pca_transf )

pcaData <- plotPCA(dds_vst_cenbrain , intgroup = c("Traveled_Dist_last", "Traveled_Dist_last1"), returnData =TRUE)

pca_centralBrain <- ggplot(data = pcaData, aes(x = PC1, y = PC2,label =name)) +

geom_point(data = subset(pcaData, Traveled_Dist_last <= 1500), aes(x = PC1, y = PC2, color = Traveled_Dist_last), size=3) +

geom_point(data = subset(pcaData, Traveled_Dist_last > 1500), aes(x = PC1, y = PC2, fill = Traveled_Dist_last1), size=3) +

geom_label_repel(label.size = 0)+

# geom_text(aes(label=name), vjust=3, size=4,nudge_x = 3, nudge_y = 3)+

ggtitle("PCA Traveled distance last run")+

xlab("PC1: 30% variance")+

ylab("PC2 : 21% variance")+

scale_color_gradient(low ="grey", high = "darkblue")+

# scale_fill_manual(aes(x = PC1, y = PC2, fill = Traveled_Dist_last1), values = c("green"))+

theme_bw()+

theme(panel.border = element_blank(),

panel.grid.major = element_blank(),

panel.grid.minor = element_blank(),

axis.line = element_line(colour="black"),

legend.position = "bottom",

legend.text=element_text(size=12),

axis.title = element_text(size = 12),

axis.text = element_text(size = 12))

pca_centralBrain

# Testing the correctness of the test runs in the central brain expression #

dds_Corrprop_test_mean <- DESeqDataSetFromMatrix(countData = centralbrain_counts, colData = indepVar_19 , design = ~ Corrprop_test_mean )

keep <- rowSums(counts(dds_Corrprop_test_mean) >= 10) >= 6

dds_Corrprop_test_mean <- dds_Corrprop_test_mean[keep,]

# A reduced model without the last distance

dds_Corrprop_test_mean <- DESeq(dds_Corrprop_test_mean, test="LRT", reduced = ~ 1)

LRT_results_Corrprop_test_mean <- results(dds_Corrprop_test_mean)

LRT_results_Corrprop_test_mean_sig <- subset(LRT_results_Corrprop_test_mean , padj < 0.05)

LRT_results_Corrprop_test_mean_sig # 0 gene "affected"

#### Optic lobes Gene expression ####

opticlob <-read.xlsx("~/Data/Supl_tables.xlsx", sheet = 5, header = T)

colnames(opticlob) <- c("Gene_ID" , "OL_605_C_2B", "OL_617_C_7Y", "OL_638_C_0W", "OL_638_C_1W", "OL_638_C_7W", "OL_638_C_BB", "OL_647_C_5W", "OL_650_C_1G", "OL_650_C_1Y", "OL_605_C_2P", "OL_650_C_3R", "OL_650_C_3Y", "OL_653_C_BR", "OL_654_C_0B", "OL_654_C_9B", "OL_654_C_9W")

rownames(opticlob) <- opticlob$Gene_ID

opticlob_counts <- opticlob[,-c(1)]

# Drop sample "CB_617_C_7Y", never drunk honey

opticlob_counts <- opticlob_counts[,-c(2)]

indepVar_15 <- indepVar[rownames(indepVar) %in% colnames(opticlob_counts),]

#Order the rows of the design data in the same order as the columns on the count data

opticlob_counts <- opticlob_counts[,order(colnames(opticlob_counts))]

colnames(opticlob_counts)

rownames(indepVar_15)

# CEnter and scale traveled distance from the last run

indepVar_15$sTraveled_Dist_last <- as.vector(scale(indepVar_15$Traveled_Dist_last, center = TRUE, scale = TRUE))

indepVar_15$Traveled_Dist_last1 <- "> 1500"

indepVar_15$Traveled_Dist_last1[indepVar_15$Traveled_Dist_last <= 1500] <- NA

######### Correct in the tested runs -average ####

dds_optlob <- DESeqDataSetFromMatrix(countData = opticlob_counts, colData = indepVar_15 , design = ~ Corrprop_test_mean )

dds_optlob_ltr_Corrprop_last <- DESeq(dds_optlob, test="LRT", reduced = ~ 1)

LRT_results_Corrprop_last_runs <- results(dds_optlob_ltr_Corrprop_last)

LRT_results_Corrprop_last_runs_sig <- subset(LRT_results_Corrprop_last_runs , padj < 0.05)

LRT_results_Corrprop_last_runs_sig # 228

mcols(dds_optlob_ltr_Corrprop_last)$maxCooks <- apply(assays(dds_optlob_ltr_Corrprop_last)[["cooks"]], 1, max)

cooksCutoff <- qf(.99, 2, 15 - 2)

sum(mcols(dds_optlob_ltr_Corrprop_last)$maxCooks > cooksCutoff, na.rm = T) #882

LRT_results_Corrprop_last_runs$padj[mcols(dds_optlob_ltr_Corrprop_last)$maxCooks > cooksCutoff] <- NA

LRT_results_Corrprop_last_runs_cooks_sig <- subset(LRT_results_Corrprop_last_runs , padj < 0.05)

LRT_results_Corrprop_last_runs_cooks_sig # 81 DEGs

LRT_results_Corrprop_last_runs_cooks_sig$gene <- rownames(LRT_results_Corrprop_last_runs_cooks_sig)

#

#visualization

# DESeq2 creates a matrix when you use the counts() function

## First convert normalized_counts to a data frame and transfer the row names to a new column called "gene"

normalized_counts <- counts(dds_optlob_ltr_Corrprop_last, normalized=T) %>%

data.frame() %>%

rownames_to_column(var="gene")

### Extract normalized expression for significant genes from the OE and control samples (2:4 and 7:9)

norm_OEsig <- normalized_counts %>%

dplyr::filter(gene %in% LRT_results_Corrprop_last_runs_cooks_sig$gene)

### Set a color palette

heat_colors <- RColorBrewer::brewer.pal(6, "YlOrRd")

### Run pheatmap using the metadata data frame for the annotation

pheatmap::pheatmap(norm_OEsig[2:16],

color = heat_colors,

cluster_rows = T,

show_rownames = F,

border_color = NA,

fontsize = 10,

scale = "row",

fontsize_row = 10,

height = 20)

LRT_results_Corrprop_last_runs_tb <- LRT_results_Corrprop_last_runs %>%

data.frame() %>%

rownames_to_column(var="gene") %>%

as_tibble()

LRT_results_Corrprop_last_runs_tb <- LRT_results_Corrprop_last_runs_tb %>%

dplyr::mutate(threshold_OE_pos = padj < 0.05

& log2FoldChange > 0)

LRT_results_Corrprop_last_runs_tb <- LRT_results_Corrprop_last_runs_tb %>%

dplyr::mutate(threshold_OE_neg = padj < 0.05

& log2FoldChange < 0)

LRT_results_Corrprop_last_runs_tb <- LRT_results_Corrprop_last_runs_tb %>%

mutate(color = case_when(threshold_OE_neg ~ "Negative",

threshold_OE_pos ~ "Positive"))

## Create an empty column to indicate which genes to label

LRT_results_Corrprop_last_runs_tb <- LRT_results_Corrprop_last_runs_tb %>% dplyr::mutate(genelabels = "")

## Sort by padj values

LRT_results_Corrprop_last_runs_tb <- LRT_results_Corrprop_last_runs_tb %>% dplyr::arrange(padj)

## Populate the genelabels column with contents of the gene symbols column for the first 10 rows, i.e. the top 10 most significantly expressed genes

LRT_results_Corrprop_last_runs_tb$genelabels[1:10] <- as.character(LRT_results_Corrprop_last_runs_tb$gene[1:10])

ggplot(LRT_results_Corrprop_last_runs_tb) +

geom_point(aes(x = log2FoldChange, y = -log10(padj), colour = color), size = 0.5) +

geom_text_repel(aes(label = genelabels, x = log2FoldChange, y = -log10(padj))) +

xlab("log2 FoldChange") +

ylab("-log10 FDR") +

geom_hline(yintercept =1.30103, linetype = "dashed")+

scale_color_manual(values = c("blue", "red", "grey")) +

theme_classic()+

theme(legend.position = "none",

plot.title = element_text(size = rel(1.5), hjust = 0.5),

axis.title = element_text(size = rel(1.25)))

pcaData <- plotPCA(dds_vst_optlob , intgroup = c("Corrprop_test_mean"), returnData = T)

pcaData$name2 <- c("605-2B", "605-2P", "638-0W", "638-1W", "638-7W", "638-BB", "647-5W", "650-1G", "650-1Y",

"650-3R", "650-3Y", "653-BR", "654-0B", "654-9B", "654-9W")

pca_optlob <- ggplot(data = pcaData, aes(x = PC1, y = PC2,label =name2)) +

geom_point(aes(x = PC1, y = PC2, color = Corrprop_test_mean), size=3) +

geom_label_repel(label.size = 0)+

# geom_text(aes(label=name), vjust=3, size=4,nudge_x = 3, nudge_y = 3)+

ggtitle("PCA Optic lobes")+

xlab("PC1: 63% variance")+

ylab("PC2 : 10% variance")+

scale_color_gradient(low ="grey", high = "darkblue")+

# scale_fill_manual(aes(x = PC1, y = PC2, fill = Traveled_Dist_last1), values = c("green"))+

theme_bw()+

theme(panel.border = element_blank(),

panel.grid.major = element_blank(),

panel.grid.minor = element_blank(),

axis.line = element_line(colour="black"),

legend.position = "bottom",

legend.text=element_text(size=12),

axis.title = element_text(size = 12),

axis.text = element_text(size = 12))

pca_optlob

# A reduced model without the last distance

dds_optlob_ltr_last_dist <- DESeq(dds_optlob, test="LRT", reduced = ~ sTotal_time_drink )

LRT_results_last_dist <- results(dds_optlob_ltr_last_dist)

LRT_results_last_dist_sig <- subset(LRT_results_last_dist , padj < 0.05)

LRT_results_last_dist_sig # 27

summary(LRT_results_last_dist)

###### ###### Distance + colony #####

dds_optlob <- DESeqDataSetFromMatrix(countData = opticlob_counts, colData = indepVar_15 , design = ~ sTraveled_Dist_last + colony)

keep <- rowSums(counts(dds_optlob) >= 10) >= 6

dds_optlob <- dds_optlob[keep,]

# A reduced model without the sTotal_time_drink

dds_optlob_ltr_colony <- DESeq(dds_optlob, test="LRT", reduced = ~ sTraveled_Dist_last )

LRT_results_colony <- results(dds_optlob_ltr_colony)

LRT_results_colony_sig <- subset(LRT_results_colony , padj < 0.05)

LRT_results_colony_sig # 8

# A reduced model without the last distance

dds_optlob_ltr_last_dist <- DESeq(dds_optlob, test="LRT", reduced = ~ colony )

LRT_results_last_dist <- results(dds_optlob_ltr_last_dist)

LRT_results_last_dist_sig <- subset(LRT_results_last_dist , padj < 0.05)

LRT_results_last_dist_sig # 0
